# Association between fetal hemoglobin, lactate dehydrogenase, and disease severity in patients with sickle cell disease at Bugando Medical Centre, Mwanza, Tanzania

**DOI:** 10.1371/journal.pone.0286891

**Published:** 2024-07-15

**Authors:** Samwel Edward Kahema, Cosmas H. Mbulwa, Charles Nkubi Bagenda, Nixon Niyonzima, Enoch Muwanguzi, Tunzo L. Mcharo

**Affiliations:** 1 Mbarara University of Science and Technology, Mbarara, Uganda; 2 Morogoro College of Health and Allied Sciences, Morogoro, Tanzania; 3 Bugando Medical Centre, Mwanza, Tanzania; 4 Uganda Cancer Institute, Kampala, Uganda; 5 Jakaya Kikwete Cardiac Institute, Dar es Salaam, Tanzania; Imam Abdulrahman Bin Faisal University, SAUDI ARABIA

## Abstract

**Introduction:**

There is a wide range of clinical manifestations in sickle cell disease (SCD). Despite having the same condition, each person’s response to disease complications differs greatly. Individuals can be categorized according to the severity of their diseases to determine which group they fall into and receive the appropriate care based on their needs. The relationship between fetal hemoglobin (HbF), lactate dehydrogenase (LDH), and disease severity in Tanzania is little understood. This investigation sought to ascertain the relationship between HbF, LDH, and disease severity in SCD patients at the Bugando Medical Center.

**Method:**

This cross-sectional study was carried out on SCD patients aged 6 months and older at the Bugando Medical Center in Mwanza, Tanzania. A total of 130 SCD patients were enrolled. The clinical history and laboratory test results for SCD patients were recorded on a specially constructed patient report form.

**Results:**

The majority of participants (56.9%) were men. For the population under study, more than half (60.8%) of participants had a moderate clinical phenotype (MCP), followed by 31.5% of asymptomatic participants and 7.7% of people with severe clinical phenotypes (SCP). Participants with SCP had substantially higher levels of LDH, with a mean level of 810.97IU/L (95% CI: 559.31–1062.64) and a p-value of 0.005. The severe clinical phenotype exhibited a significantly higher mean HbF score value of 10.09% (95% CI: 7.44–13.74%) with a p-value of 0.024 when compared to the asymptomatic and moderate clinical phenotypes.

**Conclusion:**

In SCD patients with SCP compared to ACP and MCP, the HbF levels were higher, but did not show a protective effects, and LDH can be used to predict the severity of SCD.

## Introduction

Sickle cell disease (SCD), which is a significant cause of child morbidity and mortality, is most prevalent in Sub-Saharan Africa. It was first described by Herrick in 1910 on an anemic West Indian student in Grenada [1, 2].

The disease is characterized by the presence of two copies of β^S^ (SS), a gene mutation that codes for Hemoglobin S [3]. In Africa, the frequency of β^S^ (SS) is highest in low altitudes equatorial regions followed by compound heterozygous, HbSC and HbSβ^O^ that are most common in West Africa [3, 4].

It affects about 3% of all births in the continent [3], and there could be 236905 sickle cell trait births and 16695 sickle cell disease births in Uganda per year [5]. Tanzania has one of the highest annual SCD birth rates in the world, with an estimated 10000 births per year [6]. According to Ambrose et al, the prevalence of sickle cell trait and disease in Tanzania is 20% and 1%, respectively [7].

An estimated 50 to 90 percent of SCD youngsters pass away before turning five years old [6, 8]. Extrapolating from this, there could be between 150,000 and 300,000 SCD child fatalities per year, or between 5 and 10% of the region’s overall child mortality [9, 10]. In comparison to healthy children, many surviving children with SCD, as well as tens of thousands of infants born in Africa each year with different kinds of SCD, have severe morbidity [11]. Thus, early case identification and the implementation of comprehensive healthcare management are critical for improving SCD management.

## Methods

### Study design

This was a cross-sectional study involving sickle cell disease (SCD) patients who attended a sickle cell clinic (SCC) at Bugando Medical Centre (BMC). All SCD patients attending SCC either in steady-state or crisis who were willing to participate in the study were included in the study excluding all SCD patients with chronic conditions not associated with SCD like malignancy.

### Study area

The study was conducted at Bugando Medical Centre (BMC) which is a referral, consultant, and university teaching hospital in the United Republic of Tanzania. BMC offers a sickle cell clinic with more than 400 children enrolled [12].

### Sampling technique

To recruit participants for the study, a simple random sampling procedure was used. The selected individuals were allocated with unique identification numbers.

### Data collection tool and method

To collect information regarding the patient’s demographics and clinical history, a specific patient report form was created. Following a review of the SCD patient’s clinical history, venous blood was drawn from the chosen participants. The blood sample drawn from each participant was used to determine the amount of HbF and LDH. The data collection tool was validated in a pilot study before data collection.

### Samples processing

To determine the percentage of fetal hemoglobin (HbF) in each participant, a *Smart LifeLC*^*TM*^ High performance liquid chromatography (HPLC) machine was used. In determination lactate dehydrogenase (LDH), patient sample was processed using a chemistry analyzer, the *COBAS Integra 400 plus*.

### Data processing and analysis

Data were processed and analysed using STATA Version 12.1. The severity of SCD was assessed using the clinical score. Components of the scale that were used to calculate clinical score included the number of hospitalizations per year, number of blood transfusions per year, number of occlusive crises per year, hip disease, leg ulcers, hepatobiliary complications, neurologic events, renal disorders, and body weights for children. Clinical phenotype score was built up by recording the individual scores related to the most relevant medical history parameters. The following scores were applied to define the clinical score scale of severity: mild or asymptomatic clinical phenotype (ACP) score ≤5, moderate clinical phenotype (MCP) score between 6 and 15, and severe clinical phenotype (SCP) score of 16, and above (Table 1).

**Table 1:** Clinical score table.

Clinical criteria	Variables	Score (points)
Days of hospitalization/year	≤1	0
	2–7	2
	≥8	5
Severe vaso-occlusive crisis/year	0	0
	1–2	2
	≥3	5
Blood transfusion/year	0	0
	1–2	2
	≥3	5
Hip disease	Absent	0
	Present	5
Leg ulcer	Absent	0
	Present	5
Hepatobiliary complications	Absent	0
	Cholecystectomy	2
	Present	5
Neurologic events	Absent	0
	Present	5
Renal disorders	Absent	0
	Present	5
Children 5–19 years	≥50 percentile	0
	<50 percentile	2
Body weight Children < 5 years	≥50 percentile	0
	<50 percentile	2
Total	Asymptomatic clinical phenotype	≤5
	Moderate clinical phenotype	6–15
	Severe clinical phenotype	≥16

A table for calculation of clinical score adapted from Mikobi [13].

The proportion of sickle cell disease (SCD) severity was determined by taking the number of SCD patients category divided by the total number of participants in the study.

Analysis of variance (ANOVA) was used to study the relationship between lactate dehydrogenase (LDH) level and fetal hemoglobin (HbF) level and sickle cell disease (SCD) severity. The Chi-square test was used to determine the statistical significance of the relationship.

Standard deviation and standard error were used to describe the spread around the mean. Descriptive statistics were used to summarize the data. A *p*-value of less than or equal to 0.05 was considered significant.

### Ethical consideration

This study was conducted after ethics approval from the Mbarara University of Science and Technology (MUST) Research Ethics Committee on behalf of the Uganda National Council for Science and Technology (UNCST) in Uganda and the Bugando Medical Centre/Catholic University of Health and Allied Science (CUHAS) joint Research Ethical Committee in Tanzania. A signed, written informed consent was obtained from every participant before enrolling in this study. A full explanation of the study was given to the children’s parents/ guardians and was allowed to review the consent form which included all the relevant information required such as the purpose of the study, the procedures to be followed, and the risks and benefits of participation. For children (18 years old and below) to participate in this study, assent for the child to participate was obtained from the child and approved by parents or guardians. To maintain confidentiality, participant names were not used, instead, they were assigned a unique identification number.

## Results

### Demographic characteristics of the participants

This study had a total of 130 individuals, with males accounting for the vast majority 74(56.9%). The participants’ median age was 8 years, with interquartile range between 5 and 11 years. About three-quarters of the participants (77%) were aged 5 and above. The bulk of participants, 93 (71.5%), lived within 20 kilometers from the Bugando Medical Centre. The majority of participants 98(75.4%) were elementary and secondary school students, while the remainder had not attended school. Sukuma made up half of the participants 65 (50%) followed by Haya and Kurya 26(20%) and other tribes (30%) (Table 2).

**Table 2:** Demographic characteristics of participants.

Characteristics	All N (%)
**Age**	
Less than five years	30(23)
Five years and above	100(77)
**Sex of participant**	
Male	74(56.9)
Female	56(43.1)
**Location Relative to the clinic facility**	
In reasonable proximity (<20Km)	93(71.5)
Relatively far away(≥20Km)	37(28.5)
**Schooling**	
Yes	98(75.4)
No	32(24.6)
**Ethnicity**	
Sukuma	65(50.0)
Haya	13(10.0)
Kurya	13(10.0)
Other	39(30.0)

### Clinical characteristics of the participants

About 4 in 10 (40.8%) of the participants had abnormal weight for age based on weight for age z-score for under-five (<50^th^ percentile) and abnormal weight percentile (< 50^th^ percentile) for five years of age and above. About three-quarters, 96 (73.8%) had low fetal hemoglobin values (<10%) and the remaining proportion had higher fetal hemoglobin (HbF) levels (≥10%). The majority of 100(76.9%) had no history of prolonged hospital stay (≥8 days of admission per year). About half of the participants 63 (48.5%) had episodes of severe vaso-occlusive crises. Fifty four (54) participants (41.5%) had history of blood transfusion, and 45 (34.6%) of the participants had history of hip disease. A minority of the participants 12(9.2%) had other complications such as leg ulcers, neurologic complications, and renal disorders (Table 3).

**Table 3:** Clinical characteristics of participants.

Characteristics	All N (%)
**Weight for age**	
Abnormal (<50^th^ percentile)	53(40.8)
Normal (≥50^th^ percentile)	77(59.2)
**Fetal Hemoglobin Values**	
HbF levels (<10%)	96(73.8)
HbF levels (≥10%)	34(26.2)
**History of prolonged hospital stay (≥ 8 days admissions per year)**	
No	100(76.9)
Yes	30(23.1)
**Severe vaso-occlusive crisis**	
No	67(51.5)
Yes	63(48.5)
**Blood transfusion**	
No	76(58.5)
Yes	54(41.5)
**Hip Disease**	
Absent	85(65.4)
Present	45(34.6)
**Other complications** *	
Absent	118(90.8)
Present	12(9.2)

* Leg ulcers, hepatobiliary, neurologic, and renal disorders

### The proportion of SCD severity among patients with SCD

The majority of participants 60.8% (95% CI: 51.3–68.5) had moderate clinical phenotypes (MCP), followed by asymptomatic clinical phenotypes (ACP) at 31.5% (95% CI: 23.1–40.0), and severe clinical phenotypes (SCP) at 7.7% (95% CI: 3.8–12.3) (Table 4).

**Table 4:** The proportion of SCD severity among patients with SCD.

The severity of SCD	Frequency	Proportion, % (95%CI)
**All**		
Asymptomatic Clinical Phenotype	41	31.5(23.1–40.0)
Moderate Clinical Phenotype	79	60.8(53.1–68.5)
Severe Clinical Phenotype	10	7.7(3.8–12.3)

### The relationship between HbF and SCD severity among patients with SCD

A mean score of 7.92% for the HbF value was obtained with (95%CI: 7.10–8.73%) for the whole cohort. Participants with the asymptomatic clinical phenotype (ACP) had a mean HbF level of 9.07% (95%CI: 7.29–10.85), those with the moderate clinical phenotype (MCP) had an average mean HbF level of 7.04 (95%CI: 6.17–7.91), and those with the severe clinical phenotype (SCP) had an average HbF level of 10.09% (95%CI: 7.44–13.74). The increased value of HbF in SCP may be caused by uneven distribution of HbF among F-cells and some might have insufficient concentrations to inhibit sickle hemoglobin (HbS) polymerization [14].

The overall difference in mean HbF levels in both severity groups was significant with a P value of 0.024 (Table 5).

**Table 5:** The relationship between HbF level and SCD severity.

The severity of SCD	Frequency	HbF values %		P-value
		Mean (SD)	95%CI	
**All**				0.024
Overall	130	7.92(4.69)	7.10–8.73	
Asymptomatic Clinical Phenotype	41	9.07(5.63)	7.29–10.85	
Moderate Clinical Phenotype	79	7.04(3.88)	6.17–7.91	
Severe Clinical Phenotype	10	10.09(5.09)	7.44–13.74	

P-value computed from one-way ANOVA (p<0.05 implies statistical significance otherwise not)

### The relationship between LDH and SCD severity among patients with SCD

A high serum LDH level was observed in participants with the severe clinical phenotype (SCP). Participants with ACP had a mean LDH of 562.88IU/L (95%CI: 509.84–615.93), and those with MCP had a mean LDH of 705.845IU/L (95%CI: 640.70–770.99), and those with SCP had an average mean LDH of 810.97IU/L (95%CI: 559.31–1062.64). In comparison with study participants with ACP and MCP, study participants with SCP had a significant increase in LDH with a P-value of 0.005 (Table 6).

**Table 6:** The relationship between LDH and SCD severity.

The severity of SCD	Frequency	LDH value IU/L		P-value
		Mean (SD)	95%CI for Mean	
**All**				0.005
Overall	130	668.844(272.96)	621.48–716.21	
Asymptomatic Clinical Phenotype	41	562.88(168.07)	509.84–615.93	
Moderate Clinical Phenotype	79	705.845(290.83)	640.70–770.99	
Severe Clinical Phenotype	10	810.97(351.81)	559.31–1062.64	

P-value computed from one-way ANOVA (p<0.05 implies statistical significance otherwise not)

## Discussion

### Proportion of participants with severe SCD

In this study, the proportion of SCD severity was determined to be 7.7% for the severe clinical phenotype (SCP), 60.8% for the moderate clinical phenotype (MCP), and 31.5% for the asymptomatic clinical phenotype (SCP). The findings of this study are comparable with the findings of Cesar et al. 2019 who reported a severity proportion of severe clinical phenotype (SCP) of 9%, in contrast to the proportions of asymptomatic clinical phenotype and moderate clinical phenotype, which were 57% and 34%, respectively [15] and this could be attributed to the limited availability of disease-modifying medications in Tanzania [16, 17].

Again, the findings of this study contradict those of Adeodu et al.’s study in Nigeria, which found that only 69.5% of participants had an asymptomatic clinical phenotype, 30.5% had a moderate clinical phenotype, and none had a severe clinical phenotype [18]. The cause for this variation could be Tanzania’s limited supply of disease-modifying medications [16, 17, 19].

### Relationship between LDH and SCD severity

The findings of this study showed that LDH levels were higher in SCD patients, even those with asymptomatic clinical characteristics. The mean blood LDH level among SCD patients in this study was 668.84IU/L, which is nearly double the recognized standard limits (240-480IU/L). These findings corroborate those of Ballas and Stojanovic and Lionnet who discovered higher serum LDH in SCD patients even in mild instances. This could be related to ongoing red blood cell breakdown (hemolysis) and other tissue damage induced by vaso-occlusion [20, 21].

Furthermore, the serum LDH level was substantially higher in SCD patients with the severe clinical phenotype (810.97IU/L) compared to moderate and asymptomatic clinical phenotypes, 705.84IU/L and 562.88IU/L, respectively, in this study. When compared to ACP, the mean LDH levels in the SCP and MCP groups were considerably greater. The findings of this study accord with those of Borsu and Mikobi who found greater serum LDH levels in SCD patients with severe clinical phenotypes (SCP) compared to those with asymptomatic and moderate clinical phenotypes [13, 22]. The high LDH levels in SCP may be attributed to significant intravascular hemolysis, ischemia-reperfusion damage, and tissue death [21].

### Relationship between HbF and SCD severity

The differences in mean HbF levels in both severity groups in this study were significant with a P value of 0.024. In the SCP group, the mean HbF level was higher compared to ACP and MCP groups. Patients with SCP may have higher amounts of HbF in some cases because HbF may be distributed unevenly among F-cells and some cells may have insufficient concentrations to prevent HbS polymerization [14, 23].

In this study, the protective effects of HbF in patients with SCD was not observed. This is contrary with the findings by Adeodu and Odenheimer in which participants with asymptomatic clinical phenotype had higher levels of HbF supporting the protective postulation of HbF against SCD severity [18, 24]. The findings of this study are also contradictory to those of Mpalampa in which greater HbF levels had a protective effect in SCD patients [25].

## Conclusion

Lactate dehydrogenase (LDH) levels was significantly higher in sickle cell disease (SCD) patients with severe clinical phenotype (SCP) compared to those with asymptomatic clinical phenotype (ACP) and moderate clinical phenotype (MCP), and therefore LDH had a great utility in predicting severity of SCD. In this study, Fetal hemoglobin (HbF) level did not show utility in prediction SCD severity as it was disproportionate between the three severity groups contrary to many studies around the world where high HbF levels demonstrated protective effects against SCD severity. Due to small number of participants (10) in the SCP group compared to ACP and MCP, 41 and 79 respectively, we see it is difficult to generalize the findings that HbF is not protective against SCD severity to the general population.

We recommend that a study with a reasonable number of SCD patients be conducted that would come out with generalizable results on the protective effects of HbF against SCD severity.

## Supporting information

S1 Dataset (XLSX)
